# Effectiveness and safety of mycophenolate mofetil and rituximab combination therapy for immune idiopathic myopathies

**DOI:** 10.1186/s13075-024-03310-z

**Published:** 2024-04-03

**Authors:** Corrado Campochiaro, Nicola Farina, Giacomo De Luca, Veronica Batani, Giorgia Trignani, Davide Vignale, Anna Palmisano, Marco Matucci-Cerinic, Lorenzo Dagna

**Affiliations:** 1 Unit of Immunology, Rheumatology, Allergy and Rare Diseases, San Raffaele Scientific Institute, Via Olgettina 60, 20132 Milan, Italy; 2 Vita-Salute San Raffaele University, Milan, Italy; 3 Unit of Radiology, San Raffaele Scientific Institute, Milan, Italy

**Keywords:** Mycophenolate mofetil, Rituximab, Myositis, Combination

## Abstract

**Introduction:**

Idiopathic inflammatory myopathies (IIM) represent a rare and heterogenous group diseases, and their treatment is not fully defined yet. According to previous small case series, the combination of mycophenolate mofetil (MMF) and rituximab (RTX) may be effective in controlling difficult-to-treat patients. Our aim was to further explore the efficacy and safety of this combined approach in patients with IIM.

**Methods:**

Patients with IIM treated with the RTX/MMF combination in our Center were retrospectively identified. After the start of combination therapy, the efficacy was evaluated at 12 months (T12) according the 2016 ACR/EULAR response criteria for IIM. Cardiac imaging and pulmonary function tests were used to monitor disease activity in patients with myocarditis and interstitial lung disease, respectively. Adverse events were recorded over the follow-up period.

**Results:**

Among the 20 patients (median age 61 years; 70% female) included in the study, anti-synthetase syndrome was the most prevalent IIM subgroup (60%). At treatment start, muscle, heart, and lung were the most commonly actively affected organs. After 12 months, a moderate or major response was observed in all patients, and creatine kinase was significantly decreased (*p-value* = 0.012). Cardiac imaging and enzymes monitoring showed a reduction of heart inflammation, while pulmonary function tests improved in patients with lung involvement. No severe side effects were observed.

**Conclusion:**

Our data show that combination of RTX and MMF is effective and safe in patients with severe and refractory IIM. Therefore, this combined treatment might represent a feasible approach for difficult-to-treat IIM cases.

**Supplementary Information:**

The online version contains supplementary material available at 10.1186/s13075-024-03310-z.

## Introduction

Idiopathic inflammatory myopathies (IIM) constitute a diverse array of disorders marked by muscle weakness that may also affect skin, joints, lungs, heart, and gastrointestinal tract [1]. The management of patients with IIM is challenging due to the clinical heterogeneity and rarity of this group of diseases. Furthermore, no guidelines based on robust medical data stemming from clinical trials are available [2].

Patients who are either refractory to or dependent from glucocorticoids can be treated with a wide array of conventional immunosuppressants (methotrexate, azathioprine, and mycophenolate mofetil [MMF]) [3]. Targeted biological treatments such as the anti-CD20 monoclonal antibody rituximab (RTX) have also proven effective in the treatment of difficult-to-treat IIM [4]. Recently, intravenous immunoglobulins have been demonstrated to be effective especially in dermatomyositis [5].

Of note, the combination treatment with MMF and RTX may be particularly beneficial in refractory cases of IIM. Few favorable reports have been published in regard to the efficacy of this therapeutic combination in IIM [6–12]more data are necessary to confirm the safety and efficacy of this approach. The aim of this study was to report the efficacy and safety of the MMF/RTX combination in a large monocentric cohort of IIM patients.

## Methods

### Study population

Adult patients with IIM, classified according to the 2017 EULAR/ACR criteria [13]followed-up at the *Myositis Clinic* of the San Raffaele Hospital in Milano, were retrospectively identified. Patients treated with MMF/RTX combination therapy and followed-up for at least 12 months after the first RTX infusion were included in the analysis. Exclusion criteria comprised toxic, viral, metabolic, endocrine, inclusion-body, and cancer-associated myopathies. All enrolled patients provided specific informed consent.

### Demographic, disease, and treatment features

The following data were obtained for each patient at RTX start (T0), which followed MMF initiation in all cases:

- Demographic variables: sex, ethnicity, and age;
- Clinical features: details about the type of IIM [1], autoantibody profile (ANA and autoantibodies against Jo1, PL7, PL12, EJ, SRP, Mi2, MDA5, TIF1γ, KU, PM-Scl100, Scl70, SSA/Ro52), disease duration, and active inflammatory involvement (i.e., muscle, lung, heart, joint, skin), as defined in the Supplementary Material;
- Previous immunosuppressive treatment;
- Characteristics of the combined MMF/RTX treatment.

### Efficacy and safety

The efficacy of the combined MMF/RTX treatment was assessed at 12 months after RTX start (T12) using the 2016 ACR/EULAR criteria for minimal, moderate, and major clinical response for IIM [14]. Variations of each International Myositis Assessment and Clinical Studies Group (IMACS) [14] core set measure between T0 and T12 was analyzed.

In patients with heart and/or lung (which were the two most commonly extra-muscular domains in our cohort, as shown in the *Results* section) involvement, modifications in troponin T levels and pulmonary function tests (forced vital capacity [FVC] and diffusion lung CO capacity [DLCO]) were evaluated. Cardiac magnetic resonance (CMR) imaging was revised to monitor response of heart inflammation to combination therapy.

Adjustments of MMF and RTX regimens and of the concomitant steroid dose, as well as adverse events were recorded over the 1-year follow-up period.

## Results

**Fig. 1:**
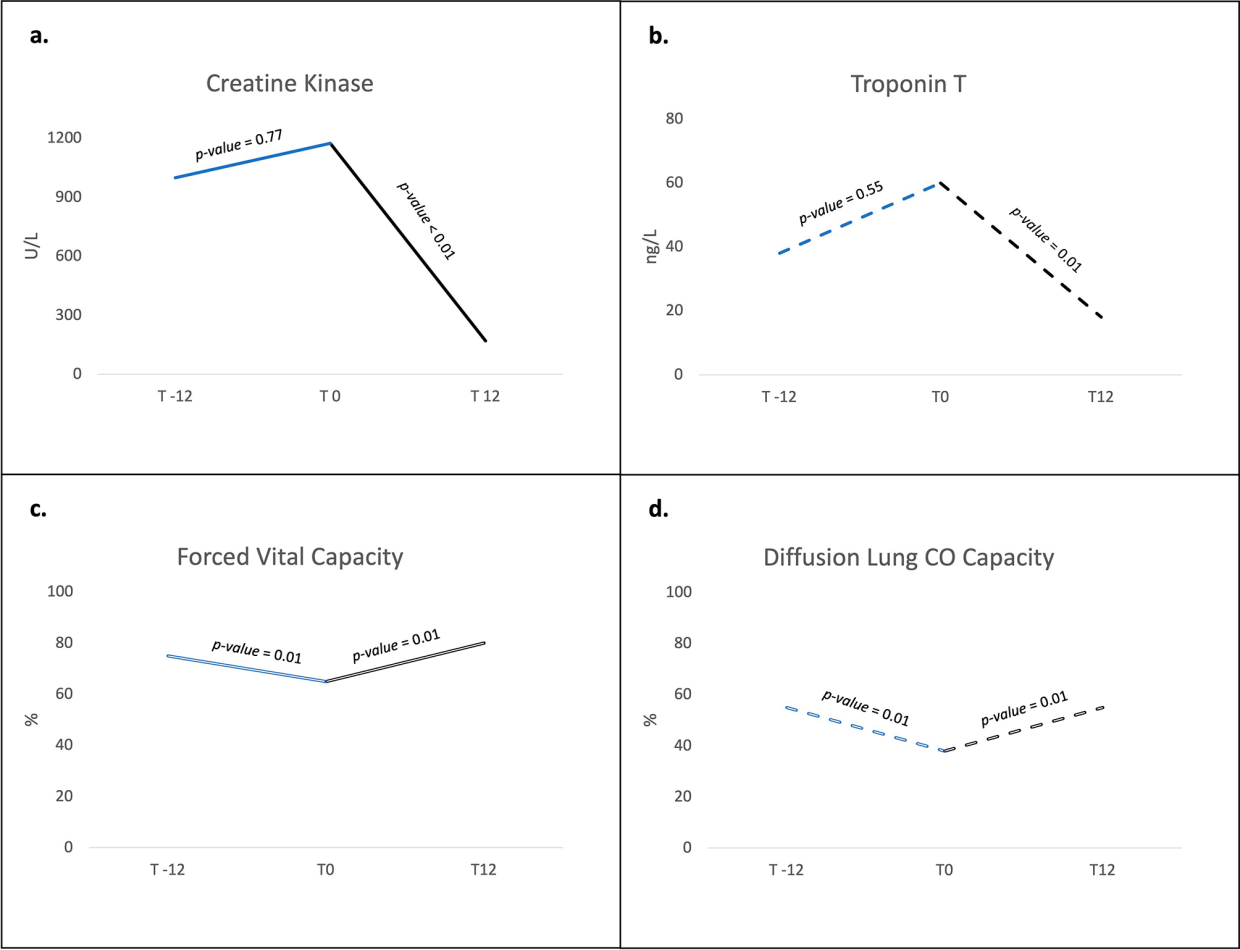
Variations of creatine kinase (**a**) and troponin (**b**) levels, as well as forced vital capacity (**c**) and diffusion lung CO capacity (**d**). T -12, 12 months before the start of combination treatment; T 0, start of combination treatment; T 12, 12 months after the start of combination treatment

In the present study, 20 patients were included: their baseline demographics, clinical, and treatment features are reported in Table 1. In our cohort, anti-synthetase syndrome was the most common subgroup. Two patients had an overlap disease with systemic sclerosis, and 1 with rheumatoid arthritis. Thirteen (65%) patients were ANA-positive; the prevalence of myositis -specific and -associated antibodies profiles is reported in Fig. 1S. Two patients were seronegative.

**Table 1:** Demographic, disease, and treatment features of the study population at the start of combination treatment

Variable	Study population (*n* = 20)
Age (years)	61 (50–71)
Female sex	14 (70%)
Caucasian ethnicity	17 (85%)
Inflammatory idiopathic myopathy subgroup	
Anti-synthetase syndrome	12 (60%)
Overlap myositis	3 (15%)
Dermatomyositis	2 (10%)
Polymyositis	2 (10%)
Necrotizing myositis	1 (5%),
Disease duration (months)	23 (10–118)
Concomitant glucocorticoid therapy	
Prevalence	17 (85%)
Median daily dosage (mg)	8.75 (5-12.5)
Previous immunosuppressants	
Methotrexate	8 (40%)
Calcineurin inhibitors	6 (30%)
Azathioprine	5 (25%)
Cyclophosphamide	4 (20%)
Intravenous immunoglobulins	3 (15%)
Previous or current inflammatory involvement	
Muscle	16 (80%)
Lung	15 (75%)
Heart	7 (35%)
Joint	6 (30%)
Skin	3 (15%)

Continuous and categorial variables are reported as median values (interquartile range) and absolute numbers (percentage), respectively

In all cases, MMF/RTX combination therapy was started – after a median number of one (IQR 1–2) immunosuppressant – due to persistent or relapsing disease activity. The main indication for combination treatment start was myositis in 12 (60%) patients, myocarditis in 7 (35%), interstitial lung disease in 5 (25%), arthritis in 4 (20%) and skin rash in 2 (10%). Seven patients had two or more concomitantly active disease domains.

As far as heart involvement is regarded, all 7 patients had myocarditis, which was subclinical in 5 and arrhythmic in 2 cases. Non-specific interstitial pneumonia was the most common pattern of lung involvement observed in our patients (*n* = 13, 80%), followed by organizing pneumonia and usual interstitial pneumonia (*n* = 1, 7%, each). Five patients met the criteria for progressive pulmonary fibrosis at T12 [15].

MMF was always started at 1 g/day and then up titrated to the daily dosage of 2 or 3 g, depending on disease severity. RTX was added with a median delay of 9.5 (IQR 4.5-26.25) months since MMF start. All patients received two RTX cycles (at T0 and after six months), each composed of two infusions of 1 g administered two weeks apart.

At T12, a moderate clinical response was observed in 8 (40%) cases, with 12 (60%) patients showing a major response. No cases of minimal or absent responses were recorded.

Each IMACS set core measure significantly improved from T0 to T12 (Table 1S and Fig. 1a). A significant reduction in levels of troponin T was observed in patients with myocarditis in this timeframe (Fig. 1b). This was accompanied by an improvement in CMR findings as shown in Table 2S and Fig. 2S. Both FVC and DLCO significantly improved in patients with interstitial lung disease (Fig. 1c and d).

Median daily dose of concomitant glucocorticoid therapy decreased from 8.75 (IQR = 5-12.5) mg at T0 to 0 (IQR = 0–5) mg at T12 (*p-value* = 0.003). Only 8 (40%) patients were still on steroid at the end of the study period. The daily dosage of MMF was decreased over the follow-up period to 2 g in 17 (85%) patients and 1 g in 3 patients (15%) patients.

Infections were reported in 10 (50%) patients over the 12-month follow-up period. Six patients had upper respiratory tract infection, 2 had urinary tract infections, 1 had gastroenteritis, and 1 had herpes simplex virus labial infection. None of these patients required hospitalization nor permanent discontinuation of treatment.

One patient developed Bell’s paralysis after the first administration of RTX but fully recovered and could be safely retreated at T6 without further episodes. After the second RTX cycle, 1 patient developed late-onset neutropenia which was not complicated by infection and did not require hospital admission. This was the only case in which RTX was discontinued and therefore combination treatment stopped – MMF was safely continued instead.

## Discussion

Our data show that the MMF/RTX combination is effective and safe in patients with IIM, and, as far as we know, our cohort is the largest described to date to this regard.

In patients with IIM, both MMF and RTX are effective as monotherapy and their use has increased over the years [3, 4]. These drugs help dampening both muscle inflammation and extra-muscular manifestations of IIM, such as interstitial lung disease and myocarditis [16]. In light of their effectiveness, MMF and RTX have more recently been used in combination in patients with difficult-to-treat IIM. According to few case reports [6–12], this therapeutic strategy may lead to favorable results in refractory patients, even in case of life-threatening involvement. The same approach has also been successfully evaluated in the treatment of interstitial lung disease in a trial including a minority of patients with IIM [17]. However, data about the efficacy and tolerability of this combined approach cannot be generalized based on such studies as they involved a small cumulative number of patients.

Our study on a monocentric cohort of 20 patients with IIM confirmed the efficacy of the MMF/RTX combination as all our patients obtained a significant response one year after treatment. In fact, each clinical and biochemical IMACS core set measure had significantly improved at this timepoint. Beside the clear benefit on muscle inflammation – as shown by the reduction in clinical scores and CK levels – the MMF/RTX combination also resulted in a significant improvement in extra-muscular disease activity.

In addition to interstitial lung disease, which is a well-known complication of IIM [1], our patients had a remarkable prevalence of myocarditis, which is in line with what our group previously reported in a larger cohort [16]. This finding may be linked to the systematic diagnostic algorithm that we implement in clinical practice, as detailed in the *Supplementary Material* and our previous work [16].

A sequential monitoring based on pulmonary function tests and CMR, as well as troponin levels, showed that both lung and heart involvement – which are linked to increased mortality in patients with IIM [18] – responded well to the combination treatment. This is in line with the efficacy shown for this pharmacological approach in IIM [6–12], as well as other connective tissue diseases [19–21].

RTX has historically been used in patients with seropositive autoimmune diseases [22]. In fact, the anti-CD20 antibody has previously shown particular effectiveness in patients with IIM and positive autoantibodies, especially in patients with antisynthetase syndrome [23]. Accordingly, previous successful reports about the use of a combined MMF/RTX therapy have only involved seropositive patients with IIM (5 with anti Jo1 antibodies and 2 with anti-MDA5 antibodies). While our cohort was predominantly composed of patients with antisynthetase syndrome, we described the efficacy of combination therapy in a wider spectrum of myositis -specific and -associated autoantibodies, as well as in seronegative patients. Hence, neither the presence of specific biomarkers nor the specific myositis subgroup necessarily influences the potential benefit related to the MMF/RTX combination.

Despite the concomitant administration of two immunosuppressive agents, no severe adverse events were recorded over the follow-up period, which is in line with previous reports [6–12]. Late-onset neutropenia was the only adverse events that led to RTX discontinuation, though it was not complicated by any infection. Of note, none of our patients, as per our clinical practice, was treated with prophylactic antibiotic or antiviral therapies. Interestingly, the overall infectious burden of MMF/RTX combination therapy was low in our patients, and in no case hospital admission was required. This favorable outcome may be linked to the reduction over the study period of the median dosage of steroids, which are one of the most notable drivers of infections in rheumatic patients [24].

Our study has some limitations. First, data collection was retrospective, and the treatment regimen was not standardized. Second, our cohort had a small sample size, though it is the largest published to date to this regard and is in agreement with the rarity of IIM. Third, our study population was characterized by a remarkable heterogeneity in terms of autoantibody positivity and clinical manifestations. Fourth, the follow-up period was relatively short, although one year is a relevant timepoint to evaluate treatment response.

## Conclusions

In conclusion, our study highlights the efficacy and safety of the MMF/RTX combination in patients with IIM. In our experience, this scheme not only improves muscle involvement but also internal organ inflammation, and its efficacy is not influenced by the disease subgroup. Hence, a combined MMF/RTX therapy may represent a feasible choice in patients with difficult-to-treat, severe IIM. Randomized, controlled clinical trials are mandatory to confirm our findings and determine the optimal regimen and doses.

### Electronic supplementary material

Below is the link to the electronic supplementary material.

Supplementary Material 1

## Data Availability

The datasets used and/or analysed during the current study are available from the corresponding author on reasonable request.
