# Supplementary material for: Effectiveness and safety of mycophenolate mofetil and rituximab combination therapy for immune idiopathic myopathies
Source: Arthritis Res Ther. 2024 Apr 3;26:79. doi: 10.1186/s13075-024-03310-z (PMC10988925; doi:10.1186/s13075-024-03310-z)
Supplement: Supplementary file 1 — Supplementary Material 1 [file 13075_2024_3310_MOESM1_ESM.docx]

**Effectiveness and safety of mycophenolate mofetil and rituximab combination therapy for immune idiopathic myopathies**

***Supplementary material***

**Evaluation of disease domains**

All patients included in the current study underwent, as per our internal guidelines, the following tests to detect active inflammatory involvement of different disease domains:

- Muscle: clinical evaluation and creatin kinase levels
- Lung: clinical evaluation and pulmonary function tests
- Heart: clinical evaluation, troponin T and NTproBNP levels, standard 12-leads ECG, 24-hour ECG-Holter, echocardiography
- Joint and skin: clinical evaluation

In case of suspected muscle inflammation at baseline evaluation, patient subsequently underwent electromyography, muscle MRI and, in selected cases, muscle biopsy.

In case of suspected interstitial lung disease, patients underwent chest CT.

In case of suspected heart involvement, patients underwent cardiac MRI, and, in selected cases, endomyocardial biopsy.

**Figure 1S**. Myositis -specific and -associated profiles in our study population.

**Figure 2S**. Cardiac magnetic resonance images of a patient from our cohort with myocarditis before (panels A to F) and eight months after (panels G to L) the start of combination therapy. Short-tau inversion recovery images did not reveal any areas of focal oedema both at baseline and at follow-up (F and L, respectively). However, T2 mapping at baseline (C) revealed an increase of T2 relaxation time (D) (mean 55.5 ms; normal value <50 ms), especially in the septum and inferior wall of the left ventricle, associated to an increase of native T1 values (B) at T1 mapping (A) (mean 1058 ms; normal value <1045 ms), suggesting diffuse edema. After therapy, both native T1 relaxation time (G and H) and T2 relaxation time (I and J) were reduced (mean 990 ms and 52.6 ms, respectively). Late gadolinium enhancement sequences did not reveal significant myocardial fibrosis or necrosis at both time points (E and K respectively).


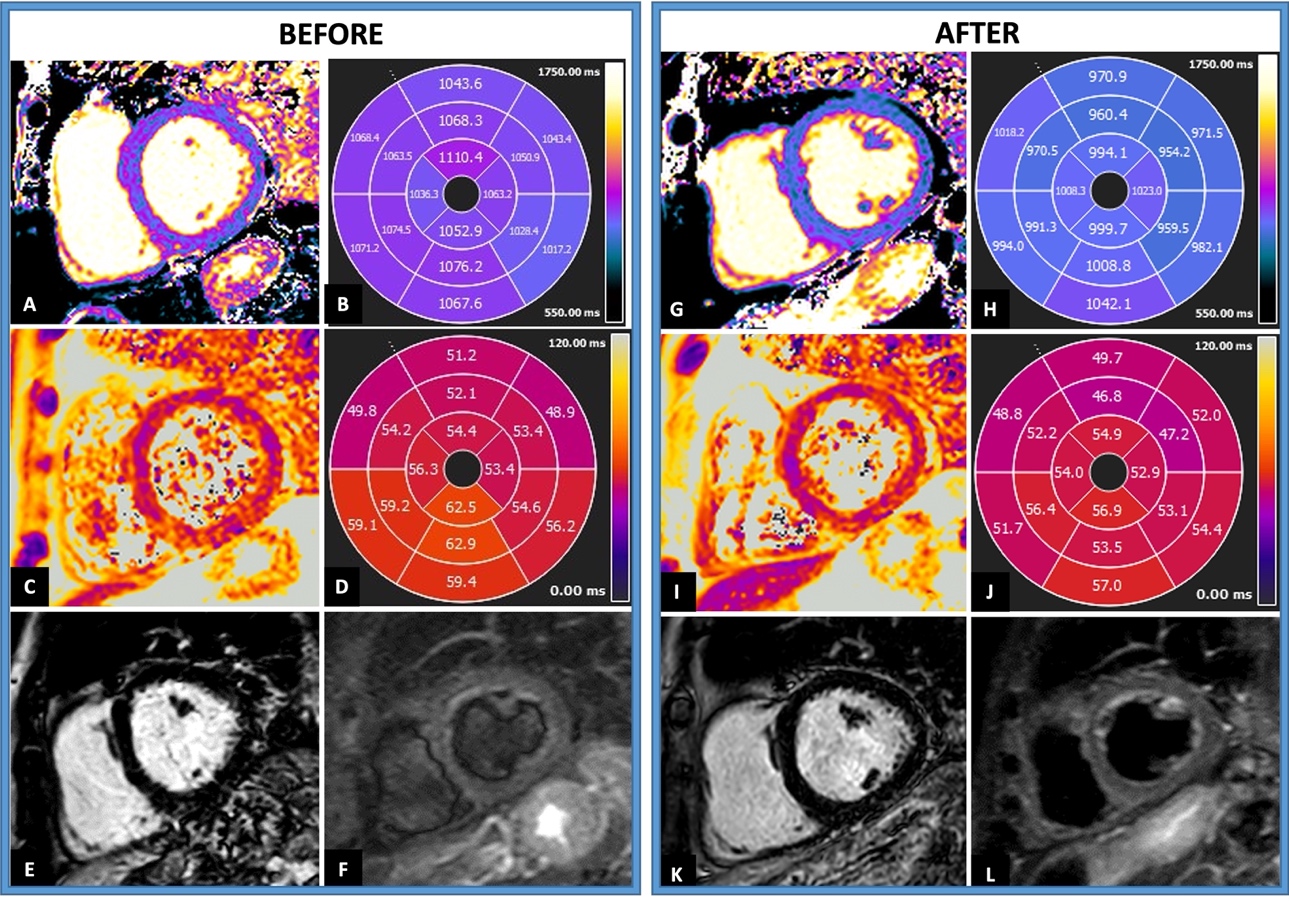


**Table 1S**. Modifications of International Myositis Assessment and Clinical Studies Group (IMACS) core set measure between the start of MMF/RTX combination treatment start (T0) and the 12-month timepoint (T12) – creatine kinase variations are reported in the main manuscript.

| Core set measures | T0 | T12 | *p-value* |
| --- | --- | --- | --- |
| VAS physician | 81 (70.5-85) | 20 (10.25-30.75) | 0.001 |
| VAS patient | 70 (55.25-77.75) | 21 (0-35) | 0.001 |
| HAQ | 0.375 (0.156-0.469) | 0 (0-0.125) | 0.002 |
| MITAX | 0.225 (0.145-0.3) | 0.1 (0.3-0.11) | 0.001 |
| MYOACT | 0.245 (0.195-0.352) | 0.07 (0.02-0.095) | 0.001 |
| MMT8 | 77 (72.5-80) | 80 (78-80) | 0.04 |

HAQ, Health Assessment Questionnaire; MMT8, Manual Muscle Testing 8; MITAX, Myositis Intention To Treat Activity Index; MYOACT, Myositis Disease Activity Assessment Visual Analogue Scale; VAS, visual analogue scale.

**Table 2S**. Relevant imaging findings at cardiac magnetic resonance in patients with myocarditis.

| Patient | T0 | T12 |
| --- | --- | --- |
| 1 | Myocardial edema | Resolved edema |
| 2 | Myocardial edema | Resolved edema |
| 3 | Myocardial edema and fibrosis | Reduced edema; persistent myocardial fibrosis |
| 4 | Myocardial edema and fibrosis | Resolved edema; persistent myocardial fibrosis |
| 5 | Myocardial edema | Resolved edema |
| 6 | Myocardial edema | Resolved edema |
| 7 | Myocardial edema and fibrosis | Resolved edema; persistent myocardial fibrosis |
